# Prenatal attachment: using measurement invariance to test the validity of comparisons across eight culturally diverse countries

**DOI:** 10.1007/s00737-021-01105-8

**Published:** 2021-02-09

**Authors:** Sarah Foley, Claire Hughes, Aja Louise Murray, Adriana Baban, Asvini D. Fernando, Bernadette Madrid, Joseph Osafo, Siham Sikander, Fahad Abbasi, Susan Walker, Bao-Yen Luong-Thanh, Thang Van Vo, Mark Tomlinson, Pasco Fearon, Catherine L. Ward, Sara Valdebenito, Manuel Eisner

**Affiliations:** 1 Centre for Family Research, University of Cambridge, Cambridge, UK; 2 Department of Psychology, University of Edinburgh, Edinburgh, UK; 3 Department of Psychology, Babes-Bolyai University, Cluj-Napoca, Romania; 4 Department of Paediatrics, Faculty of Medicine, University of Kelaniya, Colombo, Sri Lanka; 5 Child Protection Unit, University of the Philippines, Manila, Philippines; 6 Department of Psychology, University of Ghana, Legon, Accra, Ghana; 7 Health Services Academy, Islamabad, Pakistan; 8 Caribbean Institute for Health Research, The University of the West Indies, Kingston, Jamaica; 9 Institute for Community Health Research, Faculty of Public Health, Hue University of Medicine and Pharmacy, Hue University, Hue, Vietnam; 10 Department of Psychology, Stellenbosch University, Stellenbosch, South African Institute for Life Course Health Research, Department of Global Health, Stellenbosch University, Cape Town, South Africa; 11 School of Nursing and Midwifery, Queens University, Belfast, UK; 12 Research Department of Clinical, Educational and Health Psychology, University College London, London, UK; 13 Department of Psychology, University of Cape Town, Cape Town, South Africa; 14 Institute of Criminology, University of Cambridge, Cambridge, UK

**Keywords:** Maternal-fetal attachment, Pregnancy, Measurement invariance, Lower-middle income, Cross-cultural, Parity

## Abstract

Studies in high-income countries (HICs) have shown that variability in maternal-fetal attachment (MFA) predict important maternal health and child outcomes. However, the validity of MFA ratings in low- and middle-income countries (LMICs) remains unknown. Addressing this gap, we assessed measurement invariance to test the conceptual equivalence of the Prenatal Attachment Inventory (PAI: Muller, 1993) across eight LMICs. Our aim was to determine whether the PAI yields similar information from pregnant women across different cultural contexts. We administered the 18-item PAI to 1181 mothers in the third trimester (Mean age = 28.27 years old, *SD* = 5.81 years, range = 18–48 years) expecting their first infant (*n* = 359) or a later-born infant (*n* = 820) as part of a prospective birth cohort study involving eight middle-income countries: Ghana, Jamaica, Pakistan, Philippines, Romania, South Africa, Sri Lanka and Vietnam. We used Multiple Group Confirmatory Factor Analyses to assess across-site measurement invariance. A single latent factor with partial measurement invariance was found across all sites except Pakistan. Group comparisons showed that mean levels of MFA were lowest for expectant mothers in Vietnam and highest for expectant mothers in Sri Lanka. MFA was higher in first-time mothers than in mothers expecting a later-born child. The PAI yields similar information about MFA across culturally distinct middle-income countries. These findings strengthen confidence in the use of the tool across different settings; future studies should explore the use of the PAI as a screen for maternal behaviour that place children at risk.

Postpartum depression (PPD) is a key area of research in the field of maternal and child health (Field 2010), and research is increasingly recognising the crucial importance of pregnancy as a period of potential risk and opportunity for intervention. However, research on maternal psychological health has scarcely been conducted outside of high-income countries (HICs) (known as the 10/90 gap; COHRED 1990; Henrich et al. 2010). That said, reviews that have focused on research in low- and middle-income countries (LMICs) have demonstrate that prenatal stress is as strongly associated with adverse infant outcomes as in HICs (Buffa et al. 2018; Parsons et al. 2012) and that rates of PPD often exceed those reported for HICs (e.g. Fisher et al. 2012; Woody et al. 2017). Given this, it is critically important to test the universality of processes underlying maternal and infant mental health in culturally and economically diverse contexts, as outcomes differ crucially across contexts (Vidyasagar 2006).

One area of focus, as illustrated in a recent systematic review (Tichelman et al. 2019), has been the role of maternal-fetal attachment (MFA)—defined as the strength of mothers’ emotional ties with the fetus (and also known as prenatal bonding; Walsh 2010). MFA assessments capture variability in expectant mothers’ behaviours, cognitions and emotions towards the fetus, which appear important for positive prenatal health practices such as giving up smoking (e.g. Lindgren 2001), attending clinics and exercising regularly (Cinar et al. 2017). In a meta-analysis of 14 studies that both spanned prenatal and postnatal periods and included direct observational ratings of parent-infant interactions, Foley and Hughes (2018) found that MFA predicted caregiver sensitivity in interactions with their infant, which is a potent influence on children’s cognitive and socio-emotional development (Mills-Koonce et al. 2015). Thus, valid measurement of MFA has potentially far-reaching clinical implications for the health of both mothers and infants (Laxton-Kane and Slade 2002).

Predictors of MFA include gestational age (Yarcheski et al. 2009), good maternal mental health (Rollè et al. 2020), social support and first pregnancy (Tichelman et al. 2019). In contrast, MFA appears unrelated to demographics (e.g., education, age, marital status), fertility treatment, fetal defects or planned pregnancies (Tichelman et al. 2019). In a systematic review of 41 studies, Rollè et al. (2020) showed that treatment for depression can improve MFA. However, this research field is limited by its narrow focus on parents (mainly mothers) living in HICs (Brandon et al. 2009). For example, all but two of the 77 studies included in Tichelman et al.’s (2019) review were from HICs.

Of the two MFA studies with LMIC samples included in Tichelman et al.’s (2019) review, the first involved 672 mothers in rural Bangladesh. Edhborg et al. (2011) found prenatal MFA (assessed via a translation of the widely used Prenatal Attachment Inventory; PAI: Muller 1993) was positively associated with bonding when the infant was 2–3 months old and negatively associated with PPD symptoms. In the second study, Lingeswaran and Bindu (2012) assessed the feasibility of measuring MFA in India with a sample of 230 pregnant women using the Maternal-Fetal Assessment Scale (MFAS; Cranley 1981). Their findings showed average MFA scores (*M* = 87.43, *SD* = 10) were at the lower end of the range (minimum 70–maximum 114), which the authors interpreted as reflecting social and economic restrictions on women’s ability to develop a relationship with the fetus, coupled with a cultural emphasis upon birth rather than pregnancy. However, another study in India (not included in Tichelman et al. 2019) found no association between PAI scores and depression in gestational surrogates or matched controls (Lamba et al. 2018). Existing findings therefore paint a mixed picture regarding the construct validity of the PAI in LMICs. Furthermore, in both the Bangladeshi and Indian samples, the Cronbach’s alpha was lower than for English versions and included no further psychometric analysis of the questionnaires.

Punamäki et al. (2017) asked 511 expectant Palestinian mothers living in Gaza to complete the PAI. They reported that lack of social support mediated the association between war trauma and reduced MFA, but found MFA was unrelated to PPD. Given the scarcity of research on MFA in LMICs, it is difficult to judge whether this lack of association reflects the particular challenges of living in a war zone, a more general contrast in the salience of MFA across resource settings or cultural differences in the meaning or validity of the instruments used (note Punamäki et al. (2017) were unable to replicate the established PAI factor structure).

A key step in evaluating the conceptual equivalence of MFA ratings across different contexts is to construct latent factor models and test for measurement invariance across groups. That is, assessing whether a tool shows an equivalent structure and meaning across distinct groups. Theoretically, assessing whether an instrument yields similar information in different LMICs is necessary to test the cultural universality of inferences about specific causal pathways. Practically, demonstrating across-site equivalence is useful in establishing whether similar or distinct methods of identifying at-risk individuals should be used across settings.

The current study first aimed to assess the conceptual and measurement equivalence of the PAI across eight LMICs in widely different global regions. By testing for measurement invariance across eight LMICs, we aim to establish whether PAI items capture variation in how MFA is manifest across different groups. Finally, if measurement invariance is established, our next two aims were to compare mean levels of MFA across site and birth parity.

## Methods

### Participants

Trained female fieldworkers invited women attending antenatal appointments to participate in Evidence for Better Lives Study – Foundational Research (EBLS-FR), a prospective birth cohort study in eight different sites (*N* = 1208) that represent distinct social and cultural conditions across major world regions: Koforidua (Ghana, *n* = 150), Kingston (Jamaica, *n* = 152), Tarlai Kalan (Pakistan, *n* = 150), Valenzuela (Philippines, *n* = 154), Cluj-Napoca (Romania, *n* = 150), Ragama (Sri Lanka, *n* = 152), Worcester (South Africa, *n* = 150) and Hue (Vietnam, *n* = 150). Inclusion criteria were as follows: (i) third trimester of pregnancy (i.e. weeks 29–40), (ii) aged over 18 and (iii) living primarily within the study’s defined geographical area. The average recruitment rate across sites was 82%. Table 1 presents sample characteristics by site for the 1181 women expecting singleton pregnancies in the current study (excluding 27 multiple pregnancies). On average, mothers were 28.27 years old, *SD* = 5.81 years, range: 18–48 years (note expectant mothers in Jamaica, Pakistan and South Africa were significantly younger than mothers in Romania, Ghana, Sri Lanka and Vietnam). For 359 /1181 (30%), this was their first pregnancy (note half of the Romanian mothers were experiencing their first pregnancy whilst Pakistan had the smallest proportion of women experiencing their first pregnancy across sites). The sample was diverse in terms of level of education: 4% none, 11% primary, 48% secondary, 16% vocational and 21% university (lowest to highest level of maternal education, respectively: Pakistan, Ghana, Jamaica, South Africa, Vietnam, Sri Lanka, Philippines and Romania).

**Table 1:** Site-specific sample demographics

	Ghana *n* = 146	Jamaica *n* = 151	Pakistan *n* = 143	Philippines *n* = 152	Romania *n* = 148	South Africa *n* = 143	Sri Lanka *n* = 149	Vietnam *n* = 149	Between-site differences	
	*M (SD)*	*M (SD)*	*M (SD)*	*M (SD)*	*M (SD)*	*M (SD)*	*M (SD)*	*M (SD)*	*F*	*η*^2^
Age of mother (years)	29.05 (6.38)	25.57 (5.69)	27.28 (5.24)	27.63 (6.05)	30.07 (4.62)	26.89 (5.99)	29.75 (5.56)	29.93 (5.16)	13.07***	.07
Gestation (weeks)	34.35 (3.78)	33.93 (3.06)	31.82 (3.72)	32.75 (2.98)	33.16 (3.12)	34.25 (3.31)	32.38 (2.86)	33.21 (3.00)	11.18	.06
Grades passed in education system	8.33 (4.44)	10.46 (1.06)	7.80 (4.79)	11.44 (2.66)	12.80 (1.93)	10.51 (1.88)	11.83 (1.47)	10.53 (2.48)	49.67	.24
	*%*	*%*	*%*	*%*	*%*	*%*	*%*	*%*	*χ*^2^	*Cramer’s V*
First pregnancy	19.9	26.5	12.6	25	50	36.4	43	29.5	71.93	.25

***p < .001

### Procedure

Information sheets, consent forms and questionnaires were translated (using guidelines from the World Health Organization; http://www.who.int/substance_abuse/research_tools/transla- tion/en/) into the most frequently spoken languages by the participants, who provided either written or audio-recorded informed consent. Expectant mothers in their third trimester of pregnancy (*M* = 33.23 weeks gestation, *SD* = 3.36) completed the PAI and reported on demographic factors/reproductive history as part of a structured interview. The Ethics Boards of each university approved the EBLS-FR protocol (Valdebenito et al. 2020).

### Measures

#### Maternal-fetal attachment

Expectant mothers completed the 18-item PAI (Muller 1993). Participants rated how often they engaged in specific thoughts or behaviours towards the fetus (e.g., ‘*I feel love for the baby*’ or ‘*I stroke the baby through my tummy*’) on a 4-point scale: 1 = *almost never*, 2 = *sometimes*, 3 = *often*, 4 = *almost always*. A review of MFA questionnaires concluded that the PAI is psychometrically sound (e.g., Cronbach’s alpha ranges between .81 and .93) and, unlike other measures, includes behaviours *and* thoughts and feelings towards the fetus (van den Bergh and Simons 2009).

#### Background measures

Expectant mothers also reported on their age, socioeconomic status, education and previous pregnancies/ births.

### Data analysis

We analysed the data using a latent variable framework in M*plus* (Version 8.4; Muthén and Muthén 2018) and used Confirmatory Factor Analysis (CFA) to test measurement models and compare mean levels of MFA. Data screening indicated that there was no variability in responses to item 3 ‘*I enjoy feeling the baby move*’ in the Philippines and so it was removed from further analyses. Furthermore, the limited spread in the 4 response options across sites for the remaining the items indicated scores should be collapsed into dichotomous indicators (i.e., disagree/agree). This is in line with Rutkowski et al.’ (2019) recommended approach for dealing with floor/ceiling effects in response categories. Analyses applied the weighted least squares mean and variance adjusted (WLSMV) estimator. Following Muller’ (1993) original contention that MFA is unidimensional, we tested a 1-factor model alongside a 3-factor solution reflecting the dimensions of anticipation, differentiation and interaction suggested by some previous studies (e.g., Pallant et al. 2014). We evaluated model fit using three primary criteria: Comparative Fit Index (CFI) > 0.90, Tucker Lewis Index (CFI) > 0.90, Root Mean Square Error of Approximation (RMSEA) < 0.08 (Brown 2015). To test measurement invariance across site and parity, we used multigroup CFA, which involves systematically adding equality constraints to the model and testing the change in model fit of these nested models (Byrne 2012). Nested model comparisons were judged to be invariant if the CFI decreased by ≤0.020 and RMSEA increased by ≤ .003 (Svetina et al. 2020). First, we used a configural model to test for equivalence in the pattern of item loadings across site and then parity groups (i.e., the organisation of the construct is similar). If this model showed acceptable fit, we then proceeded to test metric (weak factorial) invariance, specifying that factor loadings were equal across sites (i.e., each item contributes in a similar way to the construct). If metric invariance was not achieved, we sought to improve model fit by inspecting modification indices to evaluate whether releasing item constraints would yield a partially invariant model. Next, for items that showed metric invariance, we tested for scalar (strong factorial) invariance, which involves equivalence of item thresholds (i.e., the cut-off underling the distribution of scores is consistent). Finally, if we established scalar invariance, we proceeded to test the mean differences in MFA by constraining the mean of the reference group to zero and freely estimating the other group means (Byrne 2012).

## Results

### MFA factor structure

Both the 1-factor and 3-factor solution showed an acceptable fit to the data for all sites (Table 2). However, given the high overlap between the 3 factors (*r* = > .8) we proceeded to test the across-site equivalence of the 1-factor solution.

**Table 2:** Model fit indices for a 1-factor and 3-factor model of the Prenatal Attachment Inventory by site

Site	1-factor model fit			3-factor model fit		
	RMSEA	CFI	TLI	RMSEA	CFI	TLI
Ghana	0.070	0.939	0.930	.066	.948	.939
Jamaica	0.044	0.935	0.926	.036	.957	.949
Pakistan	0.038	0.962	0.957	.036	.967	.962
Philippines	0.037	0.954	0.947	.036	.959	.952
Romania	0.086	0.829	0.804	.086	.827	.798
South Africa	0.028	0.973	0.969	.015	.992	.991
Sri Lanka	0.047	0.928	0.917	.047	.929	.917
Vietnam	0.065	0.892	0.876	.067	.888	.869

### Tests of measurement invariance across site

The configural model showed poor model fit, CFI = 0.627, TLI = 0.633, RMSEA = 0.11, SRMR = 0.061. Inspection of the modification indices suggested the removal of Pakistan would improve the model fit. Indeed, the configural model for the 7 remaining sites showed good model fit, CFI = 0.925, TLI = 0.912, RMSEA = 0.05, SRMR = 0.061. Adding metric constraints did not significantly reduce model fit: CFI = 0.921, TLI = 0.914, RMSEA = 0.05, SRMR = 0.075, but imposing constraints to examine scalar invariance did reduce model fit. Inspection of modification indices led to releasing equality constraints of factor intercepts for 15 items (items 10 ‘*I know when the baby is asleep*’, 12 ‘*I feel love for the baby*’ and 16 ‘*I stroke the baby through my tummy*’ were invariant). Thus, a partially scalar invariant model was considered to have an acceptable model fit, CFI = 0.901, TLI = 0.897, RMSEA = 0.060.

### Cross-cultural differences in prenatal attachment

Using the partial scalar-invariant model, we tested for mean differences in the latent factor across sites. We adopted a Bonferroni correction and adjusted the alpha level (i.e., *p* < .007) to account for multiple comparisons. With Ghana as the reference site, we found mean between-site differences in MFA, with lower levels in Vietnam, *b* = −.50, *z* = −3.78, *p* < .001, and higher levels in Sri Lanka, *b* = .73, *z* = 3.00, *p* < .001, however no significant contrasts with expectant mothers in Jamaica, Romania, Philippines and South Africa.

### Parity and prenatal attachment

The model testing the equivalence of the configural model across parity showed acceptable fit, CFI = 0.911, TLI = 0.894, RMSEA = 0.053, SRMR = 0.052. Adding metric constraints led to a slight improvement in model fit, CFI = 0.912, TLI = 0.902, RMSEA = 0.051, SRMR = 0.06 and imposing constraints to examine scalar invariance did not significantly reduce model fit, CFI = 0.903, TLI = 0.902, RMSEA = 0.052, SRMR = 0.066. We subsequently compared the mean of the MFA latent factor across primiparous and multiparous women, which demonstrated higher MFA in first-time mothers: *b* = −.21, *z* = −2.19, *p* = .029.

## Discussion

Evidence for links between MFA and clinically relevant pre- and post-natal behaviours (Rollè et al. 2020; Tichelman 2019) has relied heavily on studies from a few HICs. Addressing this gap, 1181 expectant mothers completed the PAI to examine the conceptual equivalence of MFA across eight LMICs: Ghana, Jamaica, Pakistan, Philippines, Romania, South Africa, Sri Lanka and Vietnam. The PAI showed conceptual and measurement equivalence for pregnant women in seven of the eight sites, with mean levels of MFA being lowest amongst expectant mothers in Vietnam and highest amongst expectant mothers in Sri Lanka. Whilst conceptually similar across levels of parity, MFA was greater in first-time mothers.

### A Modified Measure of MFA shows Cross-Cultural Equivalence

Consistent with findings from studies that have adapted the PAI for use in non-English speaking HICs (e.g., Pavše et al. 2019), our analyses support a one-factor MFA model. Similar to previous research with the PAI in India (Lamba et al. 2018), the items contributing to MFA were dichotomised into agree/disagree options. The MFA latent factor exhibited partial measurement invariance across seven out of the eight sites—with some of item thresholds freely estimated at the scalar level of invariance. Taken together, this suggests that a simplified two-option (agree/disagree) PAI is appropriate for use across culturally diverse groups, which should increase confidence in the validity of cross-cultural comparisons, as well as in the use of the PAI in efforts to improve the health and wellbeing of women across the world (UN General Assembly 2015).

That said, the PAI did not pass the first level of equivalence testing in Pakistan. Closer inspection of item responses in Pakistan revealed a near-floor effect for seven items. For example, few expectant Pakistani mothers endorsed items such as: *I stroke the baby through my tummy* or *I imagine calling the baby by name.* This may reflect other contrasts between Pakistan and the other sites, including elevated rates of infant mortality (i.e., < 5 years of age = 74 per 1000 live births), poverty (i.e., GDP pc = $1550) and education (as highlighted in Table 1: expectant mothers in Pakistan received significantly lower levels of education than all sites aside from Ghana). The lack of item endorsement may reflect a coping strategy for the mothers. For example, in seminal ethnographic research in the Alto do Cruzeiro, a shantytown in northeast Brazil, Sheper-Hughes (1992) described how, in the context of high infant mortality, impoverished mothers inhibited attachment towards their infant after birth (e.g., not attributing human qualities towards their infants). Alternatively, prevailing belief systems may render specific items or explicit endorsement of items inappropriate. Ethnographic studies have emphasised expectant mothers in Pakistan are very discreet about their pregnancies, with open conversations both within and outside of the family seen as immodest (i.e., inferring disclosure of sexual activity) and inviting *nazar* (the evil eye of jealousy), which may harm the unborn infant (Qureshi and Pacquiao 2013; Qamar 2016). Thus, women may internalise rather than externalise their developing bond. Highlighting the importance of cultural appropriateness, Arafah et al. (2020) included four new items in the PAI to capture the MFA amongst Arabic-speaking women in Qatar (e.g., ‘*I am careful with my activities so nothing will hurt my baby*’). Future qualitative research might therefore help highlight relevant questions for pregnant women in Pakistan or whether the MFA construct lacks validity in this context.

#### Site-specific differences in MFA

Whilst we often lack good cross-cultural research, establishing the conceptual and measurement equivalence of measures across different cultures is a key pre-requisite for drawing valid group comparisons (Boer et al. 2018). Having established that the PAI shows measurement invariance across seven sites, we found lower levels of MFA in Vietnam and higher levels in Sri Lanka, than in the other five countries. One factor that might contribute to these site contrasts is variation in antenatal care. For example, in Sri Lanka, the overwhelming majority of expectant mothers use the free, comprehensive maternal healthcare package, which includes antenatal classes that encourage expectant mothers to promote specific behaviours, to seek partner support and to think about their developing infants as individuals (Hemachandra 2011). Future research is needed to unpack the, potentially diverse, origins of differences in MFA, for example cultural norms, sampling and economic factors. Our study reinforces the importance of testing the equivalence of measures in multi-site studies in order to make meaningful comparisons.

#### First-time pregnancy: a time to reflect?

First-time mothers reported higher MFA than mothers in later pregnancies. Whilst similar results studies have been reported in high- (e.g., Tichleman et al. 2019) and upper-middle-income countries (e.g. Turkey; Özcan et al. 2018), the measurement invariance tests employed in the current study increase the confidence that there is a true and meaningful effect of parity. One simple explanation is that first-time mothers have greater psychological space for thinking about their new infant than mothers who are already taking care of younger infants. Our results suggest this parity effect is culturally universal—indeed, the magnitude of this effect appears stronger in this study than in previous meta-analyses (Yarcheski et al. 2009; Tichelman et al. 2019).

#### Strengths and limitations

The inclusion of data from eight LMICs is a key strength of the study. Notably, similar studies of measurement equivalence have excluded data from LMICs due to a low response rate (e.g., Zhang 2020). That said, our samples were recruited using a non-probabilistic sampling strategy, which potentially limits the generalisability of our results to the wider population. Furthermore, although the average rate of recruitment was high (82%), there was between-site variability (i.e., ranging from 50% in Sri Lanka to 98% in Pakistan) which suggests further research is required to rule out the possibility that elevated rates of MFA reported by expectant mothers in Sri Lanka are not the result of selection bias. In addition, sample characteristics differed across sites, for example in terms of education (see Table 1). However, having established the measurement properties of the PAI as an index of MFA, future research can examine the cultural universality/specificity of these associations as well explore the use of the PAI as a screener for maternal behaviours that may place children at risk.

## Conclusions

By adapting the PAI to yield a simple but valid tool that shows cross-cultural equivalence in seven LMICs and across birth order, we hope to stimulate further work examining prenatal psychological processes underpinning maternal and infant adjustment across the first 1000 days.
