# Correction to: Prenatal attachment: Using measurement invariance to test the validity of comparisons across eight culturally diverse countries

**DOI:** 10.1007/s00737-021-01122-7

**Published:** 2021-04-15

**Authors:** Sarah Foley, Claire Hughes, Aja Louise Murray, Adriana Baban, Asvini D. Fernando, Bernadette Madrid, Joseph Osafo, Siham Sikander, Fahad Abbasi, Susan Walker, Thang Vo Van, Bao-Yen Luong-Thanh, Mark Tomlinson, Pasco Fearon, Catherine L. Ward, Sara Valdebenito, Manuel Eisner

**Affiliations:** 1 Centre for Family Research, University of Cambridge, Cambridge, UK; 2 Department of Psychology, University of Edinburgh, Edinburgh, UK; 3 Department of Psychology, Babes-Bolyai University, Cluj-Napoca, Romania; 4 Department of Paediatrics, Faculty of Medicine, University of Kelaniya, Colombo, Sri Lanka; 5 Child Protection Unit, University of the Philippines, Manila, Philippines; 6 Department of Psychology, University of Ghana, Legon, Accra, Ghana; 7 Health Services Academy, Islamabad, Pakistan; 8 Caribbean Institute for Health Research, The University of the West Indies, Kingston, Jamaica; 9 Institute for Community Health Research, Faculty of Public Health, Hue University of Medicine and Pharmacy, Hue University, Hue, Vietnam; 10 Department of Psychology, Stellenbosch University, Stellenbosch, South African Institute for Life Course Health Research, Department of Global Health, Stellenbosch University, Cape Town, South Africa; 11 School of Nursing and Midwifery, Queens University, Belfast, UK; 12 Research Department of Clinical, Educational and Health Psychology, University College London, London, UK; 13 Department of Psychology, University of Cape Town, Cape Town, South Africa; 14 Institute of Criminology, University of Cambridge, Cambridge, UK

**Correction to: Archives of Women's Mental Health**

**https://doi.org/10.1007/s00737-021-01105-8**

The names of the authors were incorrect.

1. Bao-Yen Luong-Thanh appears twice – please remove Yen Lương Thanh Bảo

2. Replace Yen Lương Thanh Bảo with Thang Van Vo (see below).

